# Cholinergic Crisis with Normal Serum Cholinesterase Levels due to Excessive Galantamine Ingestion: A Case Report

**DOI:** 10.31662/jmaj.2023-0170

**Published:** 2024-04-01

**Authors:** Ayaka Suzuki, Taku Mayahara, Tomohiro Katayama, Hiroyuki Arai, Kazuyoshi Matsuura, Kentaro Nagata, Eisaku Maruo

**Affiliations:** 1 Department of Emergency and General Medicine, Kobe Ekisaikai Hospital, Kobe, Japan

**Keywords:** galantamine, galantamine overdose, cholinergic crisis, serum cholinesterase level, lethal dose

## Abstract

Galantamine is a cholinesterase inhibitor employed in Alzheimer’s disease management. Cholinesterase inhibitors are associated with potential cholinergic side effects that, when severe, can result in cholinergic crises. Although crises induced by other cholinesterase inhibitors, such as distigmine and rivastigmine, have been reported, cases of galantamine-induced cholinergic crises remain undocumented. This study presents a case of cholinergic crisis triggered by galantamine overdose in an 89-year-old woman weighing 37 kg with Alzheimer’s disease history, even though her serum cholinesterase levels were normal. The patient overdosed on 264 mg of galantamine, leading to rapid deterioration, marked by restlessness, tremors, sweating, diarrhea, pharyngeal gurgling, and severe hypoxia. Upon arrival at the emergency department, the patient exhibited pinpoint pupils, compromised airway, and low oxygen saturation, necessitating immediate intubation and transfer to the intensive care unit. After 72 h, the patient successfully recovered and was weaned off mechanical ventilation, maintaining normal serum cholinesterase levels. Animal studies suggest a lethal galantamine threshold of 3 to 6 mg/kg in humans. Unlike other cholinesterase inhibitors that typically reduce serum cholinesterase levels during cholinergic crises, galantamine appears to selectively inhibit acetylcholinesterase, possibly sparing butyrylcholinesterase. This selectivity may explain the normal serum cholinesterase levels.

## Introduction

Galantamine is a cholinesterase inhibitor used in Alzheimer’s disease management. Its inhibition of acetylcholinesterase stimulates the cholinergic nervous system, thus impeding the progression of cognitive decline ^(1)^. Cholinesterase inhibitors are associated with cholinergic side effects, such as diarrhea, vomiting, increased salivation, miosis, and bradycardia. When these symptoms worsen and require respiratory and circulatory management, a cholinergic crisis can occur ^(2)^. Although cholinergic crises induced by other acetylcholinesterase inhibitors such as distigmine and rivastigmine have been reported ^(3), (4), (5), (6)^, to our knowledge, cholinergic crises caused by galantamine have not yet been documented. We herein present the first case of a cholinergic crisis resulting from an overdose of galantamine without a reduction in cholinesterase levels.

## Case Report

The patient was an 89-year-old woman weighing 37 kg with a history of Alzheimer’s disease and who was living independently. She was maintained on galantamine 12 mg/tab, two tablets daily, with caregivers visiting daily to ensure proper medication adherence. On the morning of admission, a caregiver found the patient in a lethargic state with 22 empty blister packs of 12 mg galantamine tablets beside her. Her condition rapidly deteriorated, manifesting with restlessness, tremors, sweating, involuntary defecation with diarrhea, pharyngeal gurgling sounds, and hypoxia with a peripheral oxygen saturation of 79%. Consequently, emergency medical services were sought, and she was transported to our hospital.

**Figure 1.**
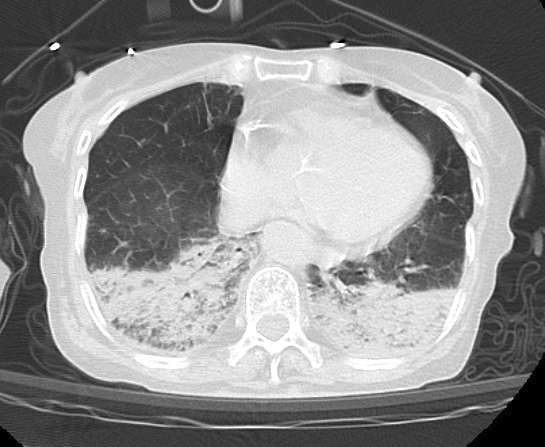
Chest computed tomography of the patient during admission revealed posterior atelectasis in both lungs, possibly due to increased airway secretions.

Upon arrival at the emergency department, the patient exhibited bilateral pinpoint pupils, sweating, excessive salivation, and airway secretions. Her vital signs were as follows: blood pressure, 180/70 mmHg; heart rate, 100 beats/min; body temperature, 36.8℃; respiratory rate, 48 breaths/min; Glasgow coma scale, 14 (E4V4M6); and peripheral oxygen saturation, 89% at 15 L of oxygen supplementation via a reservoir mask. She was immediately intubated, and positive pressure ventilation was initiated. Activated charcoal and a laxative were then administered via a nasogastric tube to inhibit the absorption of galantamine. Initial blood tests were unremarkable, and serum cholinesterase levels were within the normal range (406 U/L). Chest computed tomography revealed posterior atelectasis in both lungs (Figure 1). She was subsequently transferred to the intensive care unit (ICU), where the excessive salivation and airway secretion gradually resolved within a few hours. Mechanical ventilation was discontinued approximately 72 h later. Her serum cholinesterase levels remained normal throughout her 6-day stay in the ICU, with values of 294, 217, and 278 U/L on Days 2, 4, and 6, respectively. After rehabilitation, she was discharged without complications.

## Discussion

The patient ingested 264 mg of galantamine in a single ingestion, which is equivalent to 11 days of the maximum recommended dose. Because there are limited reports of galantamine overdose, it is extremely challenging to assess the risk associated with ingesting 264 mg of galantamine based on previous literature. Although there is no precise data regarding the lethal dose of galantamine in humans, it can be extrapolated from the lethal doses observed in single-dose toxicity studies conducted on experimental animals. According to the pharmaceutical interview form, the lethal oral doses of galantamine in mice, rats, and dogs are 36, 36, and 8 mg/kg, respectively. The human equivalent dose (HED), representing doses that produce comparable effects in experimental animals and humans, can be determined based on body surface area ^(7)^. Based on HED calculation, the estimated oral lethal dose of galantamine in humans ranges from 2.9 to 5.8 mg/kg, which is 107-215 mg for our patient weighing 37 kg. Exceeding the calculated lethal dose by ingesting 264 mg of galantamine is likely to induce severe symptoms.

**Figure 2.**
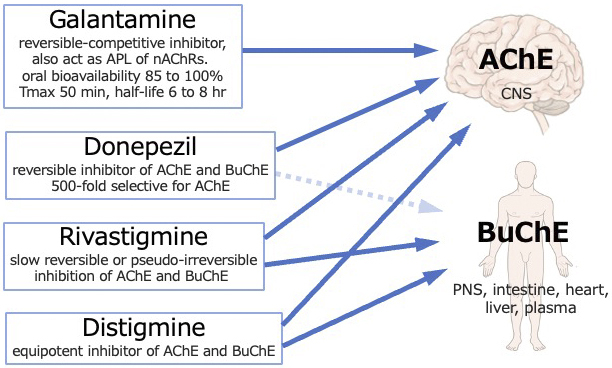
AChE predominates in the human brain, whereas BuChE is widely distributed in PNS and other organs such as the intestine, heart, and liver. Distigmine and rivastigmine inhibit AChE and BuChE, while galantamine and donepezil selectively inhibit AChE. Blood tests measure the activity of plasma BuChE. The rapid absorption and short half-time of galantamine align well with the rapid onset and resolution of symptoms in our patient. AChE: acetylcholinesterase, APL: allosteric potentiating ligand, BuChE: butyrylcholinesterase, CNS: central nervous system, nAChRs: nicotinic acetylcholine receptors, PNS: peripheral nervous system, Tmax: time to peak drug concentration.

Case reports often describe reduced serum cholinesterase levels in cholinergic crises caused by distigmine and rivastigmine ^(3), (4), (5), (6)^. Reduced serum cholinesterase levels are a crucial feature of cholinergic crises and can occasionally provide a valuable diagnostic indicator ^(3)^. However, in our case, the serum cholinesterase levels were within the normal range. Two distinct forms of cholinesterase are present, namely, acetylcholinesterase, which is primarily located in the nervous system and responsible for neurotransmission, and butyrylcholinesterase, which is detected in the serum, although its precise physiological role remains unclear ^(1)^. Cholinesterase inhibitors primarily target acetylcholinesterase in the central nervous system, while serum cholinesterase measures butyrylcholinesterase activity. Distigmine and rivastigmine inhibit both acetylcholinesterase and butyrylcholinesterase, thereby decreasing serum cholinesterase levels, while galantamine and donepezil selectively inhibit acetylcholinesterase (Figure 2) ^(8), (9)^. Mori et al. reported normal serum cholinesterase levels in a case of donepezil overdose and cholinergic crisis ^(10)^. The normal serum cholinesterase levels observed in our case may be attributed to the selective inhibition of acetylcholinesterase by galantamine.

## Article Information

### Conflicts of Interest

None

### Author Contributions

Ayaka Suzuki, Taku Mayahara: writing, designing, and manuscript editing.

Tomohiro Katayama, Hiroyuki Arai, Kazuyoshi Matsuura, Kentaro Nagata, Eisaku Maruo: care of patient and manuscript editing.

### Informed Consent

The patient had agreed and signed informed consent regarding publishing the case in an academic journal without exposing his identity.
